# Overexpression of GRK6 alleviates chronic visceral hypersensitivity through downregulation of P2Y6 receptors in anterior cingulate cortex of rats with prenatal maternal stress

**DOI:** 10.1111/cns.13827

**Published:** 2022-03-29

**Authors:** Yuan‐Qing Tian, Jia‐Hui Li, Yong‐Chang Li, Yu‐Cheng Xu, Ping‐An Zhang, Qian Wang, Rui Li, Guang‐Yin Xu

**Affiliations:** ^1^ Jiangsu Key Laboratory of Neuropsychiatric Diseases Institute of Neuroscience Soochow University Suzhou China; ^2^ Department of Anesthesiology Children’s Hospital of Soochow University Suzhou China; ^3^ Department of Gastroenterology First Affiliated Hospital of Soochow University Suzhou China

**Keywords:** anterior cingulate cortex, GRK6, P2Y6, prenatal maternal stress, visceral pain

## Abstract

**Aims:**

Visceral hypersensitivity is a major clinic symptom in patients with irritable bowel syndrome (IBS). Anterior cingulate cortex (ACC) is involved in processing the information of pain. Both G protein‐coupled receptor kinase 6 (GRK6) and P2Y purinoceptor 6 (P2Y6) are associated with neuroinflammation and pathological pain. The aim of this study was to investigate the interaction between GRK6 and P2Y6 in ACC in the development of visceral hypersensitivity of adult offspring rats with prenatal maternal stress (PMS).

**Methods:**

Visceral hypersensitivity was quantified by abdominal withdrawal reflex threshold to colorectal distension (CRD). The expression and cellular distribution of GRK6 and P2Y6 were determined by Western blotting, qPCR, and fluorescence immunohistochemistry. Co‐immunoprecipitation was used to evaluate the interaction between GRK6 and P2Y6.

**Results:**

The mRNA and protein levels of GRK6 were significantly decreased in ACC of PMS rats. The injection of GRK6 overexpression virus significantly attenuated visceral hypersensitivity of PMS rats. P2Y6’s mRNA level, protein level, and ratio of membrane protein over total protein expression was markedly increased in PMS rats. P2Y6 antagonist MRS2578 microinjection reversed visceral hypersensitivity of PMS rats. GRK6 overexpression significantly reduced P2Y6’s expression in membrane proteins and P2Y6’s ratio of membrane protein over total protein expression.

**Conclusions:**

These results indicate that decreased GRK6 leads to the accumulation of P2Y6 at neuron membrane in ACC, thereby contributing to visceral hypersensitivity of PMS rats.

## Graphical abstract

The present study has shown that PMS reduced GRK6 expression in the anterior cingulate cortex, and then decreased GRK6 led to an enhanced expression of P2Y6 in the neuronal cell membrane, thus eventually contributing to visceral pain of PMS offspring. Our findings suggest that the GRK6/P2Y6 signal pathway might be a promising strategy to treat visceral pain.

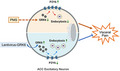

## 1 INTRODUCTION

Irritable bowel syndrome (IBS) is a common disorder of intestinal function. Due to abdominal pain or discomfort, and changes in defecation habits or stool characteristics, it creates a lot of discomfort to patients.^1^, ^2^ Visceral hypersensitivity (VH) has been suggested as one of the underlying pathophysiology of IBS. There are no obvious organic lesions or inflammation in IBS patients, and its mechanism remains largely unclear. Studies have reported that VH is a stress‐sensitive disorder of brain‐gut interactions associated with a higher prevalence of adverse early life‐forming events.^3^ We have previously reported that offspring rats with prenatal maternal stress (PMS) were afflicted with IBS‐like visceral pain,^4^, ^5^ but the central mechanism of visceral hypersensitivity (VH) in PMS rats remains largely unclear.

Studies have identified the enhanced response in the anterior cingulate cortex (ACC) to colorectal distension in visceral hypersensitivity rats,^6^, ^7^, ^8^ such as the activation of neurons, increase in transmitter release, and synaptic strengthening.^8^, ^9^, ^10^ However, the specific molecular mechanism of ACC in visceral hypersensitivity remains largely unknown. G protein‐coupled receptor kinase 6 (GRK6) belongs to the AGC Ser/Thr protein kinase family and G protein‐coupled receptor kinase subfamily. The expression of G protein‐coupled receptors (GPCRs) were downregulated due to its phosphorylation by GRKs and subsequent β‐arrestin binding.^11^, ^12^ GRKs also have a unique regulator of G‐protein signaling (RGS) domain: GTPase‐accelerating proteins that promote GTP hydrolysis by the Gα subunit of heterotrimeric G proteins, thereby deactivating the G protein and rapidly switching off GPCR signaling pathways.^13^ It was reported that downregulated GRK6 in the arcuate nucleus promotes chronic visceral hypersensitivity,^14^ but the role of GRK6 in ACC on visceral hypersensitivity was not clear.

Purinergic receptors are widely distributed in the whole nervous system, and P2Xs receptors are considered to play an important role in pain transmission.^15^, ^16^, ^17^ However, there are few studies on G protein‐coupled P2Ys receptors, and there is not enough evidence to prove whether it has an effect on visceral hypersensitivity in the central nervous system. Recently, it has been reported that the P2Y6 receptor in microglia of the spinal cord plays a role in pain regulation,^18^ but the role of P2Y6 receptors in ACC is still unclear. The P2Y6 receptor is a UDP receptor, which is coupled with G_q/11_ protein to activate phospholipase C.^19^, ^20^ It is believed that the intracellular protein level of GRKs determines the degree of desensitization and internalization of the receptors, which determines the sensitivity of GPCR.^21^ There are serine 235 (ser235) and threonine 320 (thr320) phosphorylation sites in the IL3 circular region and C‐terminal of P2Y6,^20^, ^22^ which are potential phosphorylation sites of GRK6.

Therefore, we hypothesized that PMS potentiates P2Y6 receptors via the downregulation of GRK6 in ACC, which contributes to visceral hypersensitivity of PMS offspring rats. The results of the present study may open up new avenues for preventive strategies with regard to functional gastrointestinal disorders such as IBS in adult patients.

## 2 MATERIALS AND METHODS

### 2.1 Induction of visceral hypersensitivity

**FIGURE 1:**
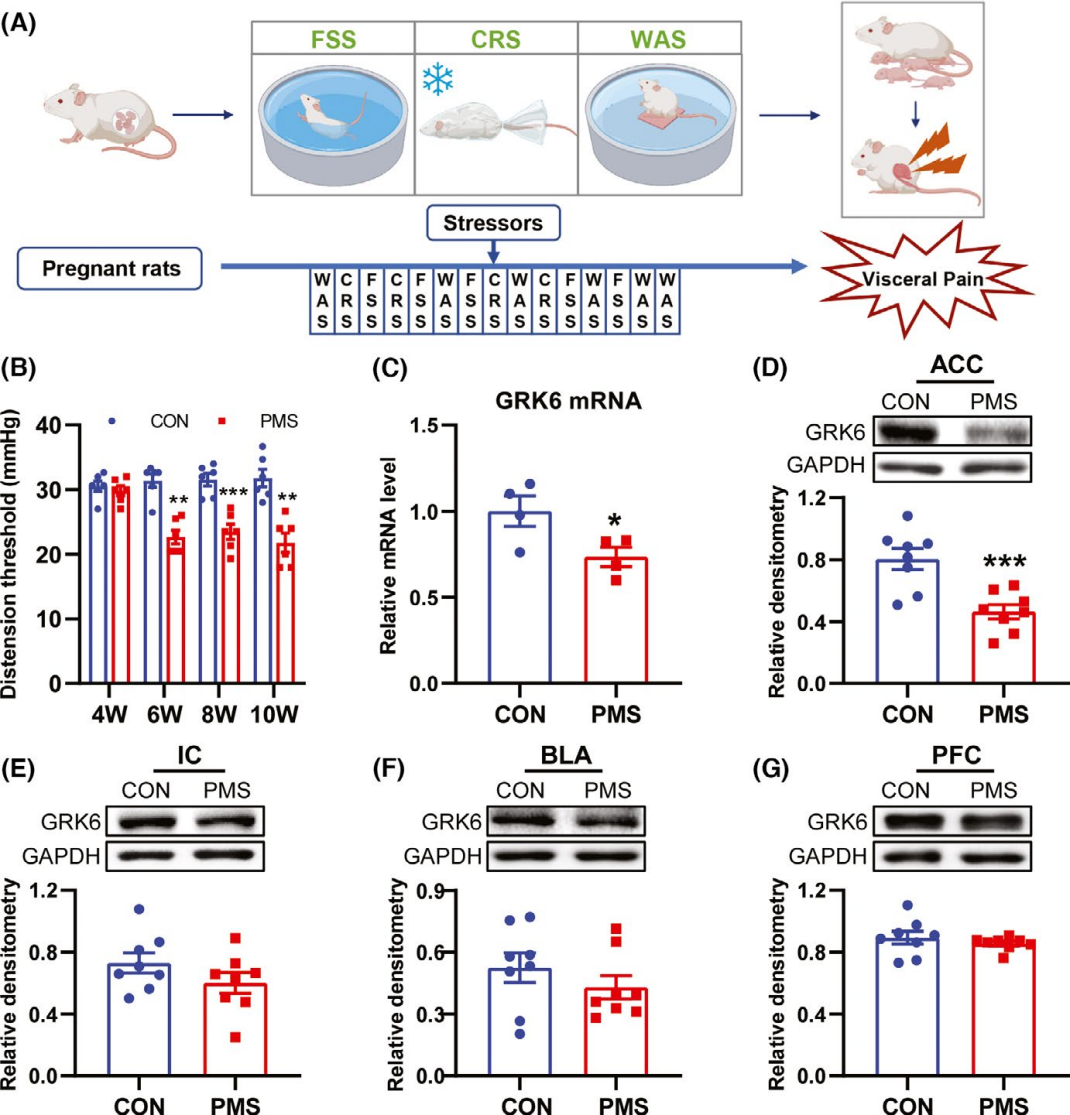
The expression of GRK6 was decreased in ACC of PMS rats. (A) Schematic representation of prenatal maternal stress procedure. (B) CRD threshold of PMS rats was significantly lower than that of CON rats at 6, 8, 10 weeks of age (**p < 0.01, ***p < 0.001, *n* = 6 for each group, two‐way ANOVA). (C) The mRNA level of GRK6 was significantly decreased in ACC of 6‐week‐age PMS rats compared with age‐matched CON rats (*p < 0.05, *n* = 4 for each group, two‐tailed two‐sample *t*‐test). (D) GRK6 protein level was markedly reduced in ACC of 6‐week‐age PMS rats compared with age‐matched CON rats (***p < 0.001, *n* = 8 for each group, two‐tailed two‐sample *t*‐test). (E–G) There was no significant difference of GRK6 expression in IC, BLA and PFC between CON and PMS rats at the age of 6 weeks (*p* > 0.05, *n* = 8 for each group, two‐tailed two‐sample *t*‐test)

All experiments were approved by the institutional animal care and use committee at Soochow University and by the Association of Laboratory Animals in Jiangsu Province, China. Adult Sprague‐Dawley rats were housed four per cage under a 12 h light/12 h dark cycle and in a temperature‐controlled room (24 ± 1°C). All rats were allowed free access to tap water and standard laboratory chow, ad libitum. The PMS model was established by stress stimulation of pregnant rats from the 7th day of gestation to delivery. The following three stress methods were randomly used each day: water avoidance stress for 60 min, cold restraint stress for 40 min, and forced swimming stress for 20 min (Figure 1A). The male offspring rats from these pregnant rats were used as PMS rats for experiments. The male offspring from untreated pregnant rats were used as control rats. Male offspring rats were used in the study to avoid the effect of estrogen on visceral pain.^23^

### 2.2 Behavioral examination of colorectal distention

Colorectal distension (CRD) threshold was detected by distention balloons to determine visceral sensitivity, as described previously.^24^, ^25^, ^26^ The balloons (length: 5 cm) were gently placed in the descending colons of rats and were secured by fixing the attached tubing to the rat's tail. Then the rats were housed in transparent resin cubicles (20 × 10 × 8 cm), and the tubing was connected to the sphygmomanometer. The value displayed by the sphygmomanometer was read when the abdomen of the rats contracted through the expansion balloon.

### 2.3 Rota‐rod test

Rats were trained at a given speed (5 to 15 rpm) for three consecutive days prior to the experiment. On the 14^th^ day after the application of lentiviral negative control (LV‐NC) and lentiviral‐GRK6 (LV‐GRK6), the time of each rat stayed on the pole at a given speed of 15 rpm was recorded. All behavioral experiments were performed in a double‐blind manner.

### 2.4 Measurement of hindpaw withdrawal threshold and latency

All rats were fed in soft padding until all the experiments finished, with the purpose of protecting rats’ feet and reducing the influence of irrelevant factors on the experimental results. Before the formal experiment, rats were drilled to acclimate, 2 days in advance. The pain threshold was measured in PMS rats before and after being injected with LV‐GRK6 and LV‐NC. Paw withdrawal threshold (PWT) in response to von Frey filament stimulation and thermal paw withdrawal latency (PWL) in response to radiant heat applied to the hindpaw plantar surface were analysed as described previously.^27^, ^28^ The PWL of each rat was recorded for 5 times (the interval between two adjacent records was 10 min), and the average value was considered as the PWL value. The cutoff time was 20 s. All behavioral experiments were performed in a double‐blind manner.

### 2.5 Drug application

To verify the effect of GRK6 and P2Y6, lentivirus‐GRK6 (LV‐GRK6) or P2Y6 antagonist MRS2578 was microinjected into the ACC (AP: +1.0 mm, ML: +0.6 mm, DV: −2.6 mm). In brief, the 1 cm wound was cut along the head centerline to expose the anterior fontanelle after anesthesia. The anterior fontanelle of the rats were aligned with the needle tip of the microsyringe fixed on the full‐automatic brain stereotaxic apparatus (RWD Life Science) and were set as the coordinate origin through software (Auto‐stereotaxic). The microsyringe needle was moved directly above the injection site after the skull site (AP: +1.0 mm, ML: +0.6 mm) was inputted by software, and following that, a small hole was drilled by the cranial perforator in the skull. The microsyringe needle was inserted slowly to 2.6 mm, and lentiviral negative control (LV‐NC), LV‐GRK6 (5×10^9^ TU/ml, 0.5 μl, Shanghai Gene Pharmaceutical Co., Ltd.), dimethyl sulfoxide (DMSO), and MRS2578 (MCE) were delivered into ACC, respectively. After the completion of the drug injection, the syringe stayed for 5 min, and finally, the needle of the microsyringe was removed slowly. CRD threshold, PWT, PWL, and Rota‐rod tests were performed and the results recorded before and after the administration of the drugs. All behavioral experiments were performed in a double‐blind manner. Molecular detection was performed 7 days after LV‐GRK6 treatment/after daily injection of MRS2578 for 7 days (10 μM, 1 μl).

### 2.6 Extraction of membrane protein and cytoplasmic protein

Membrane level and cytoplasmic level protein extractions were performed for detecting P2Y6 expression, as described previously.^29^ The separation of membrane and cytoplasmic proteins was conducted with the Mem‐PER Plus Membrane Protein Extraction Kit (Thermo Fisher Scientific) following the supplier's instructions. Briefly, after adding 300 μl of permeabilization buffer, ACC was physically disrupted by a tissue grinder and then incubated for 10 min at 4°C with constant agitation. The mixture was centrifuged (16,000 *g*, 4°C) for 15 min. The supernatant was obtained for western blotting of cytoplasmic proteins. The pellet was collected and resuspended in 100 μl of solubilization buffer for 40 min. After centrifugation (16,000 *g*, 4°C) for 15 min, the supernatant was obtained for the measurement of membrane and membrane‐associated proteins.

### 2.7 Western blotting

The expression of GRK6 and P2Y6 in ACC, prefrontal cortex (PFC), insular cortex (IC), and basolateral amygdala (BLA) of adult PMS and CON rats (6‐week‐old) were determined by western blotting. The brain tissues from CON and PMS rats were homogenized using an ultrasonic cell disrupter. The tissue solution was placed on ice for 1 h, followed by centrifugation for 30 min (20,879 *g*, 4°C). The supernatant was transferred to a new tube for protein detection. The protein concentration was estimated by the BCA protein assay kit. A total of 30 μg of the supernatant was taken in a centrifuge tube and made upto a uniform volume with normal saline. The protein loading buffer was added to the tube and the solution was denatured in 75°C water bath for 10 min. The protein solution was loaded into polyacrylamide gels (containing 10% sodium dodecyl sulfate) and fractionated on polypropylene electrophoresis (Bio‐Rad), then transferred to polyvinylidene difluoride (PVDF, Millipore) membranes at 200 mA for 2 h. After the transfer, the PVDF membranes were immersed in Tris‐HCL buffer solution containing 5% fat‐free milk at room temperature for 2 h and incubated with primary antibodies for 24 h at 4°C. After wash, the PVDF membranes were incubated with secondary antibodies for 2 h at room temperature. Primary antibodies of anti‐GRK6 (1:1000, Santa Cruz Biotechnology), anti‐P2Y6 (1:1000, Alomone Labs), anti‐P2Y6 (1:20000, Abcam), anti‐Glyceraldehyde‐3‐phosphate dehydrogenase (GAPDH, 1:2000, Goodhere Biotechnology Co., Ltd), and anti‐Sodium Potassium ATPase antibodies (1:500, Abcam) were used for detecting GRK6 and P2Y6 protein levels. Secondary antibodies of Goat anti‐rabbit IgG (1:2000; Jackson ImmunoResearch Laboratories, Inc) were used.

### 2.8 Real‐time quantitative polymerase chain reaction

The real‐time quantitative polymerase chain reaction (qPCR) was used to detect the messenger RNA (mRNA) level of GRK6 and P2Ys. Briefly, total RNA was extracted from the limbic system ACC region with trizol (Ambion). According to the system in the reverse transcription kit (Vazyme), the samples were mixed, reverse transcription reaction (85°C for 5 s and 42°C for 15 min) was carried out on the PCR apparatus (Bio‐Rad, S‐1000 Thermal Cycler), and the mRNA extracted from ACC was reversely transcribed into cDNA. Then, the mRNA expressions of GRK6 and P2Ys were detected by qPCR. Relevant conditions (1 circle at 95°C for 10 min, 40 circles at 95°C for 15 s, and 60°C for 45 s) were set on the qPCR apparatus (ABI‐7500, USA) for the onboard reaction. Melt curve analysis was used to detect different reaction products, including nonspecific products. The data were statistically analyzed according to the obtained Ct value. The sequences of the primers for GAPDH, GRK6, and P2Ys are shown in Table 1.

**TABLE 1:** Primer sequences used in the present study

Primers	Sequences (5′ to 3′)
GADPH‐F	GATGGCCTTCCGTGTTCCTA
GADPH‐R	CTTCACCACCTTCTTGATGTCATC
GRK6‐F	CCAGAAGTTTGCCACGGGTA
GRK6‐R	TCCCACAGCAATCTTGTCTACT
P2Y1‐F	GATGAATTTGAGGGCACGGC
P2Y1‐R	AAGAATGGGGTCCACACAGC
P2Y2‐F	GCTGTGCCCTTTTCCATCAT
P2Y2‐R	GTAATAGAGGGTGCGGGTGAC
P2Y4‐F	GTCATCTTCTCGGCTCCGTT
P2Y4‐R	AGGGGTCGAGTCACCTTGTA
P2Y6‐F	ACCGTGAGGATTTCAAGCGA
P2Y6‐R	AACAGGCATACAGCAGGTCC
P2Y12‐F	AACGCCTGCCTTGATCCATT
P2Y12‐R	TACATTGGGGTCTCCTCGCT
P2Y13‐F	TGCTGGGTCTCATCGCTTTT
P2Y13‐R	GGGACTCTTTAGGGACGCAC
P2Y14‐F	AAGTGGCACAAGGCGTCTAA
P2Y14‐R	GAAACAGGCGACAAATGCGA

### 2.9 Immunofluorescence assay

After anesthetia, the CON and PMS rats were intracardially perfused with 0.9% normal saline solution, (Sinopharm Chemical Reagent Co. Ltd) followed by 4% paraformaldehyde (PFA, Sinopharm Chemical Reagent Co. Ltd). The brain was moved to the tube and was fixed by 4% PFA for 3 h at 4°C. The brain was then transferred to 10%, 20%, and 30% sucrose solution (Sinopharm Chemical Reagent Co., Ltd) for gradient dehydration at 4°C. The ACC region was embedded by Tissue‐Tek O.C.T. Compound (Sakura), then cut at 20 μm on a freezing microtome (Leica). The primary antibodies used in the present experiment included anti‐rabbit‐GRK6 (1:100, Santa Cruz Biotechnology), anti‐mouse‐GRK6 (1:100, Santa Cruz Biotechnology) anti‐rabbit‐P2Y6 (1:100; Alomone Labs), anti‐mouse‐neuronal nuclei (NeuN) (1:200, Merck Millipore), anti‐mouse‐ionized calcium‐binding adaptor molecule 1 (Iba1) (1:800, Abcam), anti‐mouse‐glial fibrillary acidic protein (GFAP) (1:200, Cell Signaling Technology), anti‐mouse‐calmodulin‐dependent protein kinase II (CaMKII) (1:100, Cell Signaling Technology), and anti‐mouse‐glutamate decarboxylase 67 (GAD67) (1:100, Merck Millipore). The secondary antibodies included Alexa Fluor 488 donkey anti‐rabbit IgG (1:600, Thermo Fisher Scientific), Alexa Fluor 555 donkey anti‐mouse IgG (1:200, Thermo Fisher Scientific), and VECTASHIELD antifade mounting medium with DAPI (Vector). After staining, the images were captured by an Axioscope A1 microscope (Zeiss). The fluorescent cells were counted and analyzed using ImageJ software (Bio‐Rad).

### 2.10 Co‐immunoprecipitation (Co‐IP)

The interaction of P2Y6 and GRK6 was determined by Co‐immunoprecipitation (Co‐IP). Weak RIPA lysis buffer was added into ACC tissue, and then the ultrasonic processor was used to smash and homogenize the ACC tissue on ice with lower power (2.0 W). After letting the solution stand for 1 h, it was centrifuged, and the supernatant obtained by centrifugation was divided into three groups. In the Input group, the samples were not processed. In the IgG group and GRK6 group, agarose bead complex (Santa Cruz Biotechnology) were added to the ACC tissue for removal of nonspecific proteins. The supernatant was obtained by centrifugation for 30 min at 4°C. To remove the nonspecific proteins in the immunoprecipitation experiment, agarose beads complex was used for washing 2–3 times. GRK6 was used as bait protein to combine with the agarose bead complex, and the target protein was precipitated. In brief, in the GRK6 group, anti‐GRK6 (1:100, Santa Cruz Biotechnology) was added to the sample, followed by incubation for 2 h at room temperature with constant mixing. Then, agarose bead complex was added to the sample, followed by incubation for 12 h at 4°C temperature with constant mixing. The IgG group was taken as the negative control group, and the same amount of normal rabbit IgG (1:100, Beyotime) was added to the sample, and other conditions were unchanged. Western blotting was then performed.

### 2.11 Data analysis

All values are shown as the mean ± standard error of the mean. At first, all data in our study were tested by the normal distribution test. Two‐way repeated measure analysis of variance (ANOVA) was used to analyze behavioral data (ACC microinjection of GRK6‐lentivirus and P2Y6 antagonists). Two‐tailed two‐sample t‐test was implemented to analyze data of western blotting, qPCR, immunofluorescence and some behavioral outcomes that fit the normal distribution. Mann–Whitney test was used for non‐normally distributed data. A comparison was considered statistically significant when *p* < 0.05.

## 3 RESULTS

### 3.1 PMS decreased the expression of GRK6 in ACC

Consistent with previous studies,^4^, ^5^, ^30^ chronic visceral hypersensitivity was successfully induced in PMS offspring rats that were 6, 8 and 10 weeks of age, but no difference was observed in 4‐week‐old PMS rats when compared with CON rats (Figure 1B , *n = * 6 rats for each group, compared with CON group, **p < 0.01, ***p < 0.001, Tukey's post hoc test following two‐way ANOVA). The expression of GRK6 mRNA in ACC of 6‐week‐old PMS rats was detected by qPCR. The GRK6 mRNA level in the ACC region of PMS rats was significantly reduced when compared to that of CON rats (Figure 1C, *p < 0.05, *n* = 4 rats for each group, two‐tailed two‐sample *t*‐test). The decrease of GRK6 protein expression was further confirmed by western blotting (Figure 1D, ***p < 0.001, *n* = 8 rats for each group, two‐tailed two‐sample *t*‐test). Additionally, the protein expression of GRK6 in insular cortex (IC), basolateral amygdala (BLA), and prefrontal cortex (PFC) regions of PMS and CON rats were detected and no significant difference was observed between them (Figure 1E–G, *p* > 0.05, *n* = 8 rats for each group, two‐tailed two‐sample *t*‐test). These data indicate that GRK6 expression was downregulated in the ACC region and that GRK6 might be involved in the pain generation of PMS rats.

### 3.2 GRK6 was mainly expressed in ACC neurons

**FIGURE 2:**
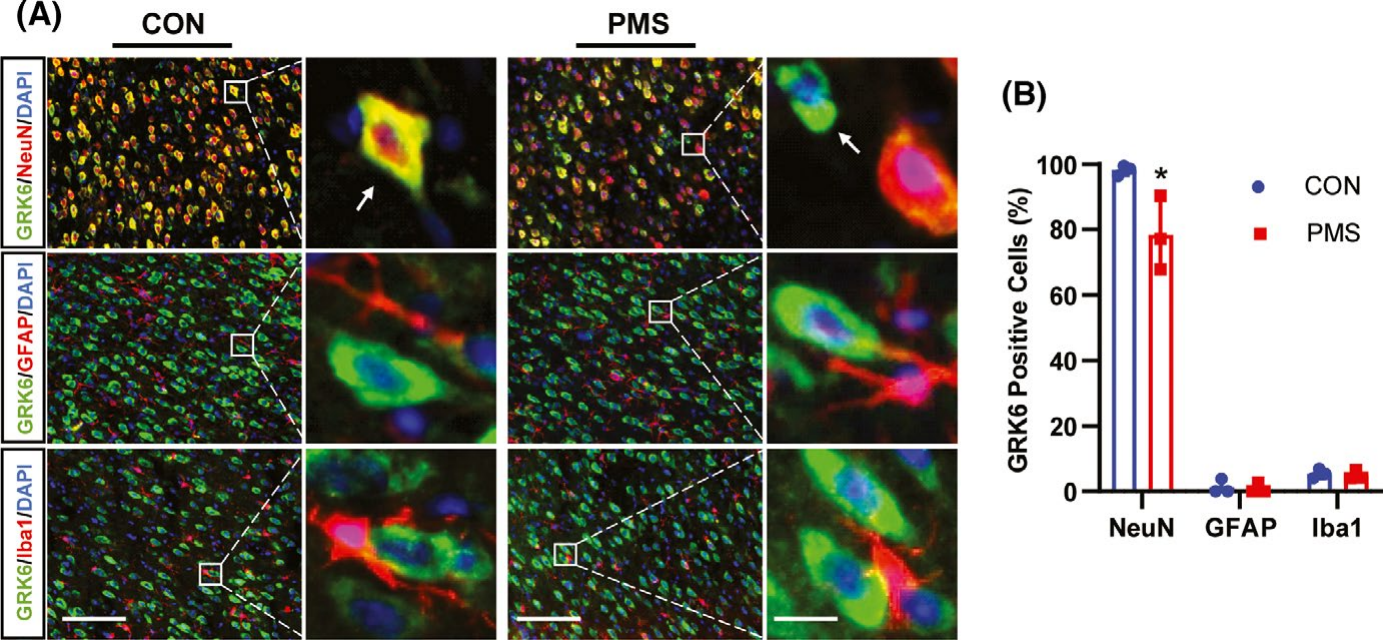
PMS reduced the percentage of GRK6‐positive neurons in ACC. (A) Immunofluorescence study showed the co‐localization of GRK6 with NeuN, GFAP, or Iba1 in ACC of CON and PMS rats (Bar = 50 μm). The enlarged pictures in the right panel were to show the extent of co‐localization of GRK6 (green) with NeuN (red), GFAP (red), or Iba1 (red) (Bar = 5 μm) and the yellow staining indicated the co‐localization. (B) Data analysis showed that GRK6 was primarily present in NeuN positive neurons with a small amount in GFAP‐positive astrocytes and Iba1‐positive microglial cells in ACC. Statistical analysis indicated that the ratio of GRK6‐positive cells in NeuN positive cells in the ACC region of PMS rats were significantly decreased when compared with CON rats (*n* = 3 for each group, *p < 0.05, two‐tailed two‐sample *t*‐test)

To determine the distribution of GRK6 in ACC, immunofluorescence assay was performed. As shown in Figure 2A, GRK6 was predominately co‐localized with NeuN (a marker of neuron), and only a small number of GRK6 was co‐labeled with GFAP (a marker of astrocytes) and Iba1 (a marker of microglia) in ACC of CON and PMS rats. Quantitative analysis showed that the percentage of GRK6 localization with NeuN, GFAP, and Iba1 was 98.15%, 1.27%, and 5.39% in CON rats, and 78.46%, 0.88%, and 4.87% in PMS rats, respectively. Statistical analysis showed that the percentage of GRK6‐positive neurons was markedly reduced in PMS rats when compared with CON rats (Figure 2B , *n* = 3 for each group, *p < 0.05, two‐tailed two‐sample *t*‐test).

### 3.3 Overexpression of GRK6 alleviated visceral hypersensitivity in PMS rats

**FIGURE 3:**
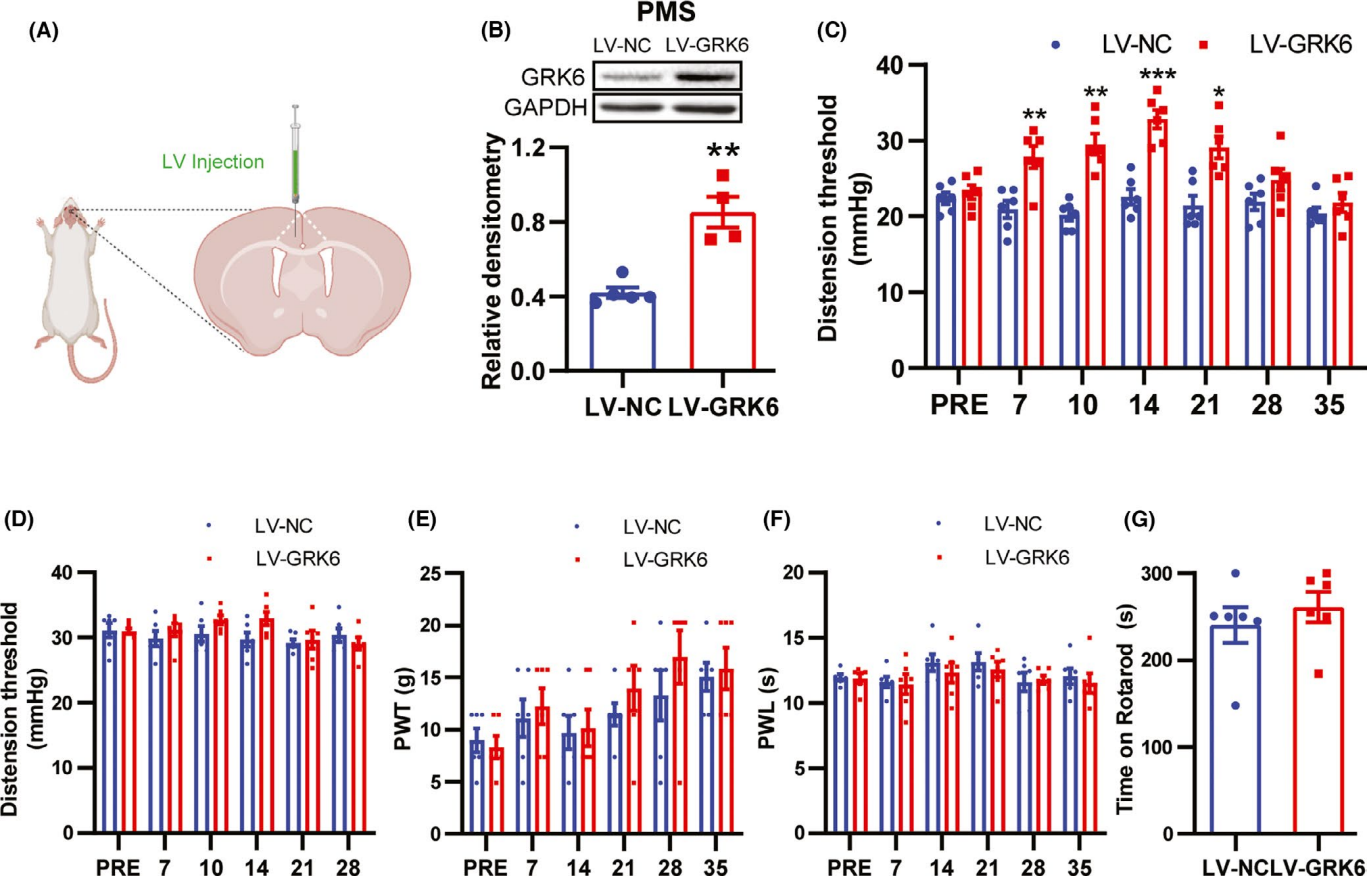
Overexpression of GRK6 in ACC region attenuated chronic visceral pain in PMS rats. (A) A schematic diagram showing the experimental design for LV injection in PMS rats ACC. (B) GRK6 protein level in ACC of LV‐GRK6 treated rats was significantly upregulated when compared with LV‐NC rats (*n* = 5 for LV‐NC group, *n* = 4 for LV‐GRK6 group, **p < 0.01, two‐tailed two‐sample *t*‐test). (C) Overexpression of GRK6 in ACC significantly elevated the CRD threshold of PMS rats from 7^th^ days to 21^th^ days after injection of LV‐GRK6 compared with LV‐NC treatment group (*n* = 6 for each group, *p < 0.05, **p < 0.01, ***p < 0.001, compared with LV‐NC, two‐way ANOVA). (D) Overexpression of GRK6 in ACC did not affect the CRD threshold of CON rats (*n* = 6 for each group, *p* > 0.05, compared with LV‐NC, two‐way ANOVA). (E–G) The time on rotarod and mechanical and thermal pain thresholds of hindpaw of LV‐GRK6 rats had no significant change between rats treated with LV‐GRK6 or LV‐NC (*n* = 6 for each group, *p* > 0.05, two‐tailed two‐sample *t*‐test)

To further investigate the involvement of downregulated GRK6 in visceral hypersensitivity of PMS rats, we overexpress GRK6 via the microinjection of lentivirus‐GRK6 (LV‐GRK6) into ACC (Figure 3A). Western blotting showed that GRK6 expression was successfully increased in PMS rats 7 days after LV‐GRK6 injection (Figure 3B, compared with LV‐NC, *n* = 5 for LV‐NC group, *n* = 4 for LV‐GRK6 group, **p < 0.01, two‐tailed two‐sample *t*‐test). Notably, GRK6 overexpression by LV‐GRK6 injection in ACC resulted in an increase in CRD threshold, indicating an analgesic effect, and the effect maintained from 7 to 21 days (Figure 3C *n* = 6 for each group, compared with LV‐NC, *p < 0.05, **p < 0.01, ***p < 0.001, Tukey's post hoc test following one‐way repeated‐measures ANOVA). We also delivered LV‐GRK6 to ACC of CON rats, and no alteration was observed (Figure 3D, *n* = 6 for each group, compared with LV‐NC, *p* > 0.05, Tukey's post hoc test following one‐way repeated‐measures ANOVA). LV‐GRK6 injection did not affect the performance of PMS rats in Rota‐rod test and mechanical and thermal pain thresholds of hindpaw (Figure 3E–G *n* = 6 for each group, compared with LV‐NC, *p* > 0.05, Tukey's post hoc test following one‐way repeated‐measures ANOVA). These results suggest that downregulated GRK6 in ACC contributes to visceral hypersensitivity of PMS rats.

### 3.4 P2Y6 expression was enhanced in ACC of PMS rats

**FIGURE 4:**
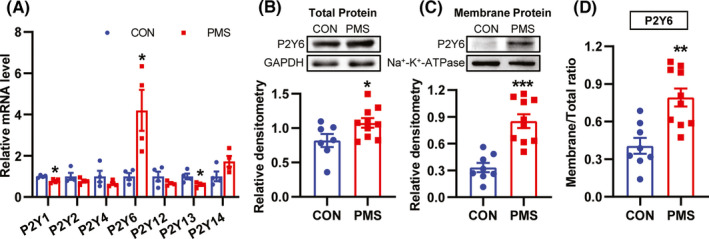
Upregulation of P2Y6 expression in ACC contributed to visceral hypersensitivity of PMS rats. (A) The mRNA level of P2Y6 was significantly increased in ACC of PMS rats compared with CON rats (*n* = 4 for each group, *p < 0.05, two‐tailed two‐sample *t*‐test). (B) Compared with CON rats, P2Y6 total protein expression was significantly increased in ACC of PMS rats (*n* = 8 for PMS rats, *n* = 9 for CON rats, *p < 0.05, two‐tailed two‐sample *t*‐test). (C) Compared with CON rats, P2Y6 membrane protein expression was significantly increased protein in ACC of PMS rats (*n* = 10 for PMS rats, *n* = 8 for CON rats, ***p < 0.001, two‐tailed two‐sample *t*‐test). (D) The ratio of P2Y6 membrane protein expression over P2Y6 total protein expression was significantly increased (Total protein level: *n* = 8 for PMS rats, *n* = 9 for CON rats; Membrane protein level: *n* = 10 for PMS rats, *n* = 8 for CON rats, compared with CON rats, **p < 0.01, two‐tailed two‐sample *t*‐test)

To evaluate the potential downstream targets of GRK6, the P2Ys expression level was measured. The mRNA level of P2Y6 was significantly increased but the expression of P2Y2, P2Y4, P2Y12, and P2Y14 was not altered (Figure 4A, *n* = 4 for each group, *p < 0.05, two‐tailed two‐sample *t*‐test). In addition, the protein expression of P2Y6 was significantly increased at both the total protein (Figure 4B, *n* = 8 for PMS rats, *n* = 9 for CON rats, compared with CON rats, *p < 0.05, two‐tailed two‐sample t‐test) and the membrane protein levels (Figure 4C , *n* = 10 for PMS rats, *n* = 8 for CON rats, compared with CON rats, ***p < 0.001, two‐tailed two‐sample *t*‐test). P2Y6 ratio of membrane protein to total protein expression was significantly increased (Figure 4D, total protein level: *n* = 8 for PMS rats, *n* = 9 for CON rats; Membrane protein level: *n* = 10 for PMS rats, *n* = 8 for CON rats, compared with CON rats, **p < 0.01, two‐tailed two‐sample *t*‐test), indicating that the transport of P2Y6 to membrane was increased. These data suggest that P2Y6 expression was upregulated in ACC and might be involved in pain generation of PMS rats.

### 3.5 P2Y6 was mainly expressed in ACC neurons

**FIGURE 5:**
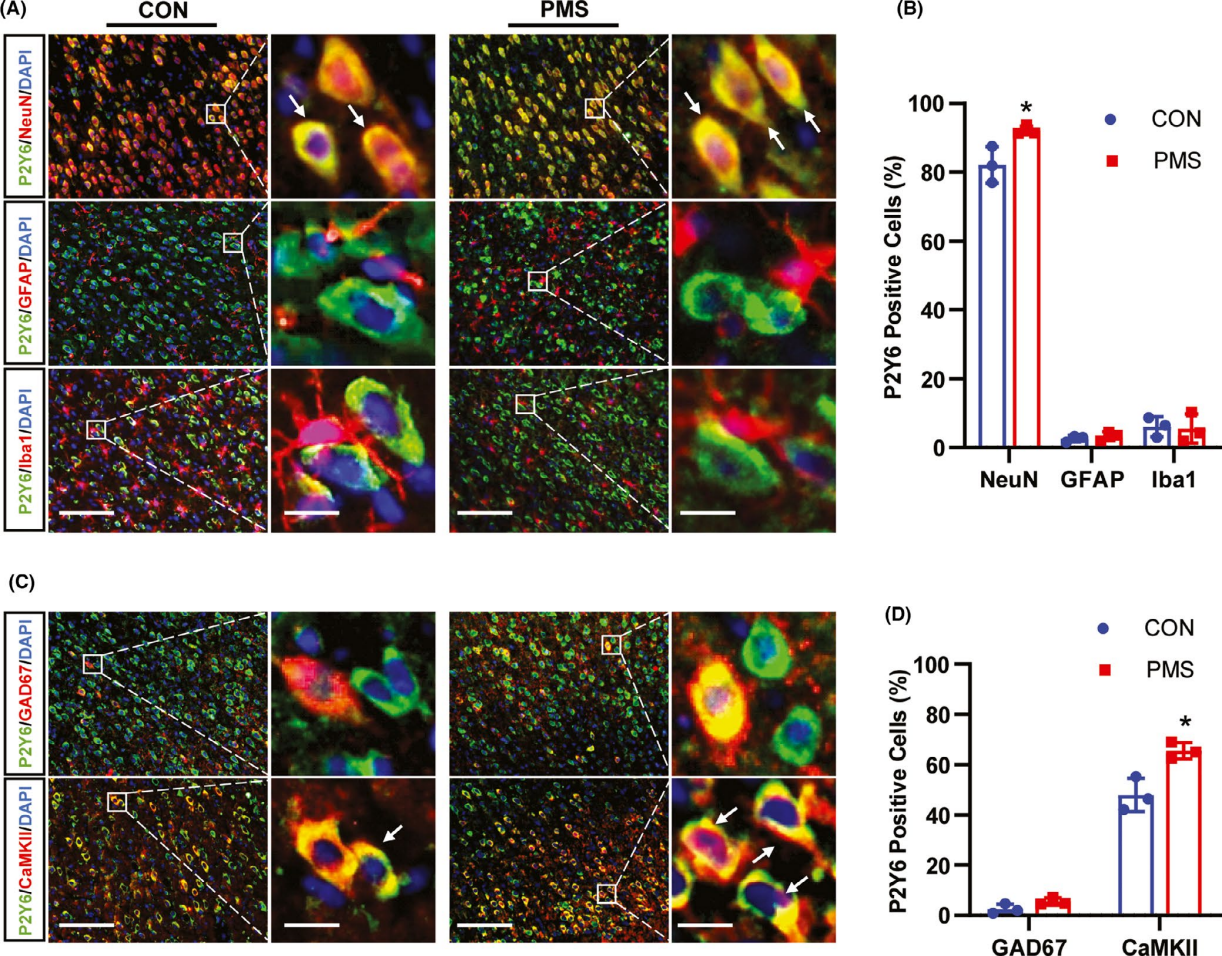
PMS enhanced the percentage of P2Y6‐positive excitatory neurons in ACC. (A) Immunofluorescence study showed the co‐localization of P2Y6 with NeuN, GFAP, or Iba1 in ACC of CON and PMS rats (Bar = 50 μm). The enlarged pictures in the right panel showed the extent of co‐localization of P2Y6 (green) with NeuN (red), GFAP (red), or Iba1 (red) (Bar = 5 μm). The yellow staining indicated the co‐localization. (B) Data analysis showed that GRK6 was mainly localized in NeuN‐marked neurons, and a small amount in GFAP‐labeled astrocytes or Iba1‐dyed microglial in ACC. The ratio of GRK6‐positive cells over NeuN positive cells in the ACC region of PMS rats were significantly increased when compared with the CON rats (*n* = 3 for each group, *p < 0.05, two‐tailed two‐sample *t*‐test). (C) Immunofluorescence assay showed the co‐localization of P2Y6 with CaMKII or GAD67 (Bar = 50 μm). The enlarged pictures in the right panel showed the extent of co‐localization of P2Y6 with CaMKII or GAD67 (Bar = 5 μm). The yellow staining indicated the co‐localization. (D) Data analysis showed that P2Y6 was mainly present in CaMKII‐labeled excitatory neurons, and a small amount in GAD67‐labeled inhibitory neurons in ACC. The percent of P2Y6‐positive cells in CaMKII‐positive cells in the ACC region of PMS rats was significantly increased when compared with the CON rats (*n* = 3 for each group, *p < 0.05, two‐tailed two‐sample *t*‐test)

It is reported that P2Y6 was mainly expressed in microglia and astrocytes.^31^, ^32^, ^33^ To confirm the distribution of P2Y6 in ACC, different cell markers were used for immunofluorescence study. Surprisingly, P2Y6 was predominately co‐localized with NeuN, but only a few with Iba1 and GFAP (Figure 5A). Quantative analysis showed that the percentage of P2Y6 localization with NeuN, GFAP, Iba1 was 82.11%, 2.57%, 6.03% in CON rats, and 92.14%, 3.41%, 5.67% in PMS rats, respectively. The percentage of P2Y6‐positive neurons was markedly increased in ACC of PMS rats when compared to that of CON rats (Figure 5B, *n* = 3, for each group, *p < 0.05, two‐tailed two‐sample *t*‐test). Further, the localization of GAD67 (a marker of GABAergic neurons) or CaMKII (a marker of excitatory neurons) with P2Y6 was detected to determine the neuron type expression of P2Y6. P2Y6 was predominately co‐localized with CaMKII but not with GAD67 (Figure 5C). Quantative analysis showed that the percentage of P2Y6 localization with GAD67 and CaMKII was 2.48%, 47.90% in CON rats, and 5.13%, 65.50% in PMS rats, respectively. The percentage of P2Y6‐positive cells in CaMKII‐positive cells was markedly increased in ACC of PMS rats when compared to that of CON rats (Figure 5D , *n* = 3, for each group, *p < 0.05, two‐tailed two‐sample *t*‐test).

### 3.6 GRK6 regulated the membrane expression of P2Y6 in ACC

**FIGURE 6:**
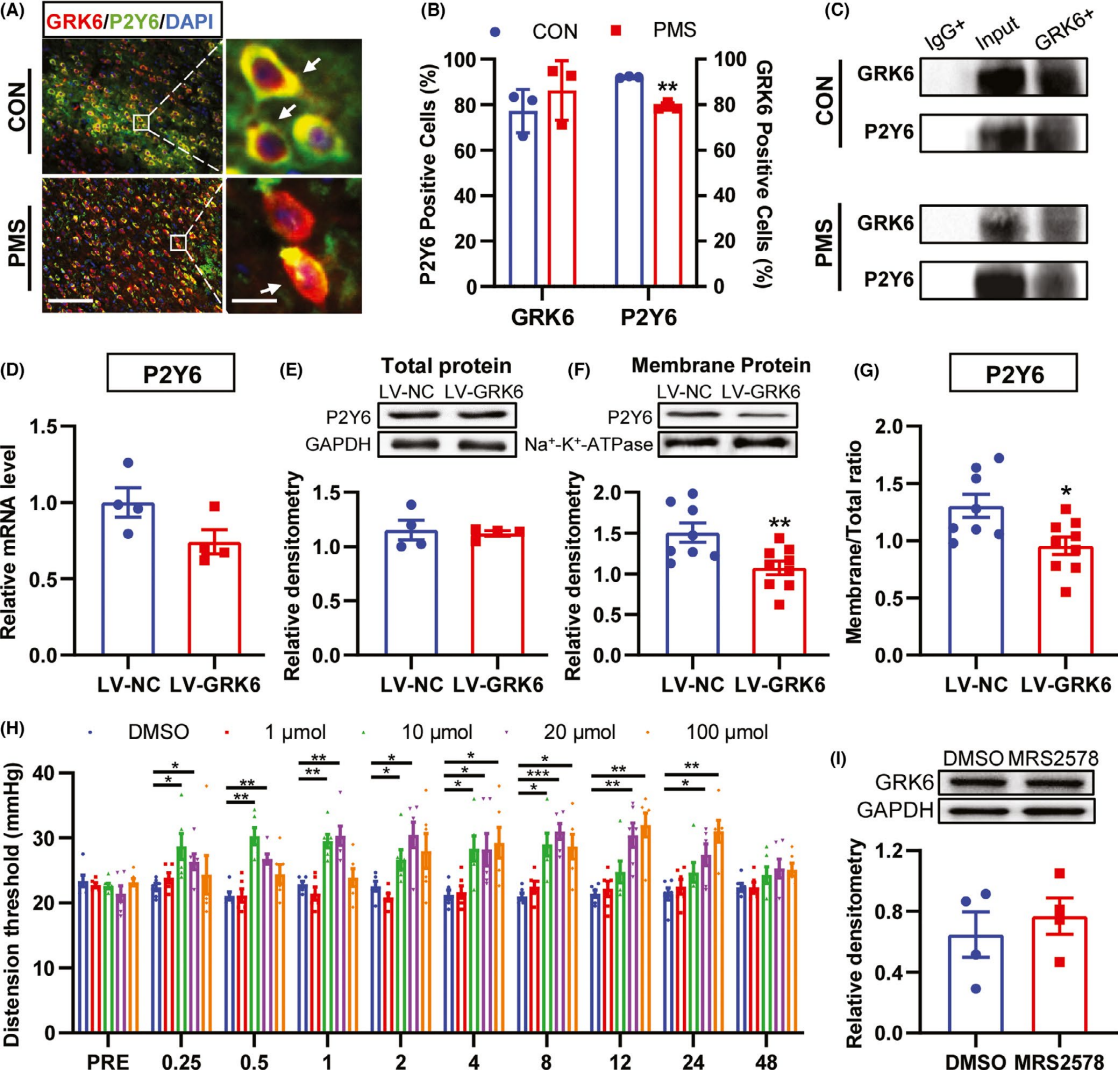
GRK6 regulated the trafficking of P2Y6 in ACC. (A) Immunofluorescence assay showed the co‐localization of GRK6 and P2Y6 in ACC (Bar = 50 μm). (B) Quantity analysis showed that the ratio of GRK6‐positive cells over P2Y6‐positive cells in the ACC region of PMS rats was significantly decreased when compared with the CON rats. There was no significant difference of P2Y6‐positive cells over GRK6‐positive cells in the ACC region of PMS rats (*n* = 3 rats for each group, **p < 0.01, *p* > 0.05, two‐tailed two‐sample *t*‐test). (C) Co‐IP study confirmed the possible interaction of GRK6 and P2Y6 in the ACC region of both CON rats and PMS rats. (D‐E) LV‐GRK6 treatment did not affect the mRNA expression and the total protein expression of P2Y6 in ACC of PMS rats (*n* = 4 for each group, *p* > 0.05, two‐tailed two‐sample *t*‐test). (F) Compared with LV‐NC rats, the membrane protein expression of P2Y6 in LV‐GRK6 group was significantly reduced (*n* = 8 for LV‐NC group, *n* = 9 for LV‐GRK6 group, **p < 0.01, two‐tailed two‐sample *t*‐test). (G) The ratio of P2Y6 membrane protein expression to total protein expression in LV‐GRK6 group was significantly decreased (total protein level: *n* = 4 for each group; membrane protein level: *n* = 8 for LV‐NC group, *n* = 9 for LV‐GRK6 group, compared with LV‐NC rats, *p < 0.05, two‐tailed two‐sample *t*‐test). (H) P2Y6 selective inhibitor MRS2578 treatment significantly increased the CRD threshold of PMS rats (*n* = 6 for each group. *p < 0.05, **p < 0.01, ***p < 0.001, two‐way ANOVA). (I) The protein level of GRK6 was not altered in ACC of PMS rats treated with MRS2578 compared with DMSO‐treated group (*n* = 4 for each group, *p* > 0.05, two‐tailed two‐sample *t*‐test)

In order to determine the relationship between GRK6 and P2Y6, immunofluorescence and co‐immunoprecipitation assays were performed. Immunofluorescence assay showed that GRK6 was co‐localized with P2Y6 in the ACC region (Figure 6A). Statistical analysis of the proportion of P2Y6‐positive cells in total GRK6‐positive cells was 77.14%, and the proportion of GRK6‐positive cells in total P2Y6‐positive cells was 92.10% in CON rats. The proportion of P2Y6 ‐positive cells in total GRK6‐positive cells was 87.21%, and the proportion of GRK6‐positive cells in total P2Y6‐positive cells was 79.14% in PMS rats. More importantly, the ratio of GRK6‐positive cells over P2Y6 positive cells in the ACC region of PMS rats were significantly decreased when compared with the CON rats. There was no significant difference of P2Y6‐positive cells of GRK6 in the ACC region of PMS rats (Figure 6B, *n* = 3, compared with CON rats, **p < 0.01, *p* > 0.05, two‐tailed two‐sample *t*‐test). Co‐IP assay showed that GRK6 interacted with P2Y6 in ACC of both PMS and CON rats (Figure 6C). In order to further determine the potential regulatory mechanism of GRK6 and P2Y6, P2Y6 mRNA and protein expression were detected on the 7^th^ day after microinjection LV‐GRK6 into ACC. LV‐GRK6 treatment did not affect the mRNA expression and the total protein level of P2Y6 (Figure 6D–E, *n* = 4 for each group, compared with LV‐NC group, *p* > 0.05, two‐tailed two‐sample *t*‐test). The membrane protein level of P2Y6 was significantly reduced after LV‐GRK6 microinjection (Figure 6F, *n* = 8 for LV‐NC group, *n* = 9 for LV‐GRK6 group, compared with LV‐NC group, **p < 0.01, two‐tailed two‐sample *t*‐test). Further analysis showed that P2Y6’s ratio of membrane protein level to total protein level significantly decreased (Figure 6G, total protein level: *n* = 4 for each group; membrane protein level: *n* = 8 for LV‐NC group, *n* = 9 for LV‐GRK6 group, compared with LV‐NC, *p < 0.05, two‐tailed two‐sample *t*‐test). To evaluate the influence of P2Y6 upregulation on visceral hypersensitivity of PMS rats, MRS2578 (a selective inhibitor of P2Y6, 1 μl) was microinjected into ACC. MRS2578 treatment significantly increased the CRD threshold of PMS rats. Moreover, the effective time of MRS2578 (10 μM) group lasted from 0.25 to 8 h (10 μM). The effective time of MRS2578 (20 μM) group lasted from 0.25 to 24 h (20 μM). The effective time of MRS2578 (100 μM) group lasted from 4 to 24 h (Figure 6H , *n* = 6 for each group, compared with DMSO, *p < 0.05, **p < 0.01, ***p < 0.001, Tukey's post hoc test following one‐way repeated‐measures ANOVA). Additionally, consecutive 7‐day microinjection of MRS2578 (10 μM, 1 μl) into ACC did not affect the expression of GRK6 (Figure 6I, *n* = 4 for each group, compared with DMSO, *p* > 0.05, two‐tailed two‐sample *t*‐test). These results indicate that GRK6 regulated the trafficking of P2Y6 in ACC cell membrane, which might contribute to visceral hypersensitivity in PMS rats.

## 4 DISCUSSION

IBS is one of the most common gastrointestinal problem managed by physicians.^34^ It is characterized by altered bowel habits and abdominal pain due to visceral hypersensitivity.^35^ The symptoms of IBS are caused by disorders of brain‐gut interactions.^36^, ^37^ The etiopathogenesis of IBS is probably multifactorial, such as early life stress, anxiety and microbiota dysbiosis. Its underlying pathophysiology remains largely unclear. Our present study provides a novel ACC mechanism involving visceral hypersensitivity of PMS rats. GRK6 downregulation in ACC neurons is involved in visceral hypersensitivity. Importantly, the overexpression of GRK6 by ACC injection of LV‐GRK6 greatly attenuated the visceral hypersensitivity of rats with PMS. This finding is consistent with the results of LV‐GRK6 injected into the arcuate nucleus of NMD rats reported previously.^14^ This confirms that the upregulation of GRK6 expression in ACC can play an analgesic role. It is therefore suggested that GRK6 possibly represents a potential strategy for the therapy of chronic visceral pain in patients with IBS.

The function of GRK6 is more likely to phosphorylate G‐protein‐coupled receptors because of its membrane‐associated nature.^38^ Here, we proved that G protein‐couple P2Y6 is a downstream molecule targeted by GRK6 under chronic visceral pain conditions. We showed that the expression of P2Y6 was upregulated both at total protein and membrane protein in ACC, suggesting that P2Y6 is involved in visceral hypersensitivity. After injection of LV‐GRK6, the total protein expression of P2Y6 was not altered, but the membrane protein expression of P2Y6 was decreased. The ratio of P2Y6 expression at membrane protein to total protein level was significantly decreased, indicating that P2Y6 internalization was increased. The above data suggest that GRK6 regulates the expression of P2Y6 on the cell membrane. Consecutive 7‐day microinjection of MRS2578 into ACC did not affect the expression of GRK6, indicating that P2Y6 could not regulate GRK6. The results of immunoprecipitation and immunofluorescence double staining of GRK6 and P2Y6 further confirmed that GRK6 likely interacted with P2Y6, suggesting that P2Y6 is a downstream molecule targeted by GRK6 under chronic visceral hypersensitivity conditions. How GRK6 regulates the transport of P2Y6 to the cytoplasm remains unclear. Previous studies have shown that there are serine 235 (ser‐235) and threonine 320 (thr‐320) phosphorylation sites in the IL3 ring region and C‐terminal of P2Y6.^20^ P2Y6 may be regulated by GRK6 phosphorylation, and the phosphorylation level of P2Y6 needs to be further proved in future research.

More importantly, the injection of P2Y6 specific inhibitor MRS2578 into the ACC can lead to the ascent of pain threshold, which proves that the decrease of P2Y6 activity alleviated visceral hypersensitivity. Notably, it has been proved that P2Y6 is involved in pain in spinal cord microglia.^39^, ^40^ Interestingly, we showed for the first time that P2Y6 is mainly expressed in ACC excitatory neurons. Recent studies have shown that P2Y6 participates in the regulation of neural function in arcuate nucleus pyramidal neurons.^41^ Therefore, it is of great value for clinical targeted cell therapy to further systematically study and differentiate the localization and function of P2Y6 in the central nervous system.

Taken together, our study provides evidence that the decrease of GRK6 in ACC promotes the expression of P2Y6 in the neuronal cell membrane, which contributes to visceral hypersensitivity. The use of LV‐GRK6 or MRS2578 in the ACC can alleviate visceral hypersensitivity of PMS rats. In the present study, we first demonstrated that P2Y6 is involved in the occurrence and development of visceral hypersensitivity in neurons, invalidating the view that P2Y6 is only involved in the mechanism of pain in glial cells. These results shed light on the mechanisms of GRK6‐P2Y6 signaling in ACC in the development of visceral hypersensitivity and provide a new therapeutic avenue into the treatment of chronic visceral hypersensitivity in patients with IBS.

## CONFLICT OF INTEREST

The author(s) declared no potential conflicts of interest with respect to the research, authorship, and/or publication of this article.

## Data Availability

The data that support the findings of this study are available from the corresponding author upon reasonable request.
